# Admission red cell distribution width–to–albumin ratio and risk of neonatal pneumonia or culture-proven sepsis in preterm infants: a retrospective cohort study

**DOI:** 10.3389/fped.2026.1855210

**Published:** 2026-08-19

**Authors:** Jiali Huang, Songbai Wang, Yuling Lin, Yongjian Zhao, Yingxiang Wang, Qinglin Rong, Xin Ye, Qiyin Cai

**Affiliations:** 1 Department of Pediatric, Jiangmen Maternity and Child Health Care Hospital, Jiangmen, China; 2 Department of Hospital Administration, Jiangmen Maternity and Child Health Care Hospital, Jiangmen, China; 3 Department of Healthcare, Jiangmen Maternity and Child Health Care Hospital, Jiangmen, China; 4 Department of Technology, Jiangmen Maternal and Child Health Hospital, Jiangmen, China

**Keywords:** composite neonatal infection, culture-proven sepsis, neonatal pneumonia, preterm infants, red cell distribution width-to-albumin ratio

## Abstract

**Background:**

Red cell distribution width–to–albumin ratio (RAR), a biomarker reflecting inflammation and nutritional status, may be associated with adverse neonatal outcomes. Given that neonatal infection remains a major complication in preterm infants, this study aimed to evaluate the association between admission RAR and the risk of subsequent neonatal infection, and to explore potential non-linear patterns.

**Methods:**

This retrospective cohort included 697 preterm infants (24⁰⁄₇–36⁶⁄₇ weeks) admitted to a tertiary NICU (2019–2024). RAR was calculated from RDW and albumin measured within 2 h of admission. The primary outcome was a subsequent composite neonatal infection during the NICU stay, defined as neonatal pneumonia and/or culture-proven sepsis. Multivariable logistic regression analyzed RAR continuously and by quartiles; restricted cubic splines and two-piecewise regression assessed non-linearity.

**Results:**

Infants in higher RAR quartiles had lower gestational age and a higher incidence of subsequent composite neonatal infection. When analyzed as a continuous variable, RAR was not significantly associated with overall neonatal infection. However, in quartile analyses, higher RAR levels were independently associated with an increased risk of subsequent neonatal pneumonia, which accounted for the majority of infection events. No significant association was observed for culture-proven sepsis. A significant non-linear relationship between RAR and pneumonia risk was identified, with an apparent inflection point at 5.65 identified in exploratory restricted cubic spline analyses. Sensitivity analyses yielded consistent results.

**Conclusion:**

Admission RAR was non-linearly associated with the risk of subsequent neonatal pneumonia in preterm infants, with an apparent inflection point at 5.65 identified in exploratory analyses. Although RAR may serve as a complementary marker for risk stratification, its performance was comparable to albumin alone without evidence of superiority over established inflammatory markers. Further prospective studies are needed to confirm these results.

## Introduction

Neonatal infection constitutes a leading cause of both morbidity and mortality for infants admitted to the neonatal intensive care unit (NICU), with preterm infants being disproportionately affected. Their heightened vulnerability stems from immature immune defenses, limited physiological reserves, and increased susceptibility conferred by frequent exposure to invasive procedures. Infectious complications in this population are associated with prolonged hospital stays, escalated needs for intensive support, and adverse long-term outcomes. Consequently, the early and accurate identification of infants at heightened risk for infection represents a cornerstone of effective neonatal care.

Inflammatory biomarkers such as C-reactive protein (CRP) and procalcitonin (PCT) are commonly incorporated into the assessment of neonatal infection. However, in preterm infants, interpretation of these markers can be challenging. Gestational age (GA)–related physiological variation, perinatal stress, and noninfectious inflammatory conditions may limit specificity, while delayed elevation after infection onset and variability in testing practices may reduce their utility for early risk assessment (1, 2). In this context, indicators derived from routinely collected laboratory data have been explored as potential adjuncts in clinical research for neonatal infection assessment.

The red cell distribution width–to–albumin ratio (RAR) is a composite index derived from red cell distribution width (RDW) and serum albumin (ALB), both routinely measured laboratory parameters. RAR is not a novel biomarker but integrates two related physiological pathways: RDW reflects impaired erythropoiesis and systemic inflammation, while ALB is a negative acute-phase reactant reduced in inflammatory states (3). By combining these measures, RAR may capture broader systemic physiological stress, reflecting inflammatory activity and altered nutritional–synthetic status (2). In adults and older pediatric populations, elevated RAR has been associated with adverse outcomes in sepsis (4), bloodstream infection (5), and other critical illnesses, serving as an adjunctive risk stratification marker rather than a specific diagnostic tool for infection.

To date, no previous studies have specifically investigated the association between RAR and neonatal infection outcomes in preterm infants, including composite neonatal infection, neonatal pneumonia, or culture-proven sepsis. While individual components of this index—such as RDW—have been examined in neonatal conditions like bronchopulmonary dysplasia and retinopathy of prematurity, the integration of RDW and ALB into a composite index has not been explored in this population. Prior research has established the prognostic value of RAR in adults and older children with critical illness (4, 6); however, its relevance for composite neonatal infection in preterm infants remains largely unknown. Accordingly, we position admission RAR strictly as an exploratory risk stratification marker in preterm infants, rather than a diagnostic biomarker. Given the distinct pathophysiology of preterm infants—characterized by immature immune responses and frequent exposure to noninfectious inflammatory stimuli—we conducted a retrospective cohort study to evaluate the association between admission RAR and the risk of subsequent composite neonatal infection (neonatal pneumonia and/or culture-proven sepsis) in preterm infants, and to explore whether this association differed according to infection subtype.

## Methods

### Study design and data source

This single-center retrospective cohort study extracted clinical data from the hospital information system (HIS) of Jiangmen Maternity and Child Health Care Hospital. All preterm infants hospitalized at this hospital between January 1, 2019, and February 29, 2024, were initially screened.

Data encompassed antenatal, perinatal, and neonatal domains, including maternal demographic characteristics, mode of delivery, GA, birth weight, laboratory parameters, and therapeutic interventions. Data extraction followed a standardized HIS-based protocol, incorporating automated retrieval and independent manual validation by two trained researchers to ensure data accuracy and completeness.

The study protocol was approved by the Ethics Committee of Jiangmen Maternity and Child Health Care Hospital (Approval No. 2026001). Owing to the retrospective design and use of anonymized data, the requirement for informed consent was waived. The study adhered to the guidelines established by the Strengthening the Reporting of Observational Studies in Epidemiology (STROBE).

### Study population and setting

**Figure 1:**
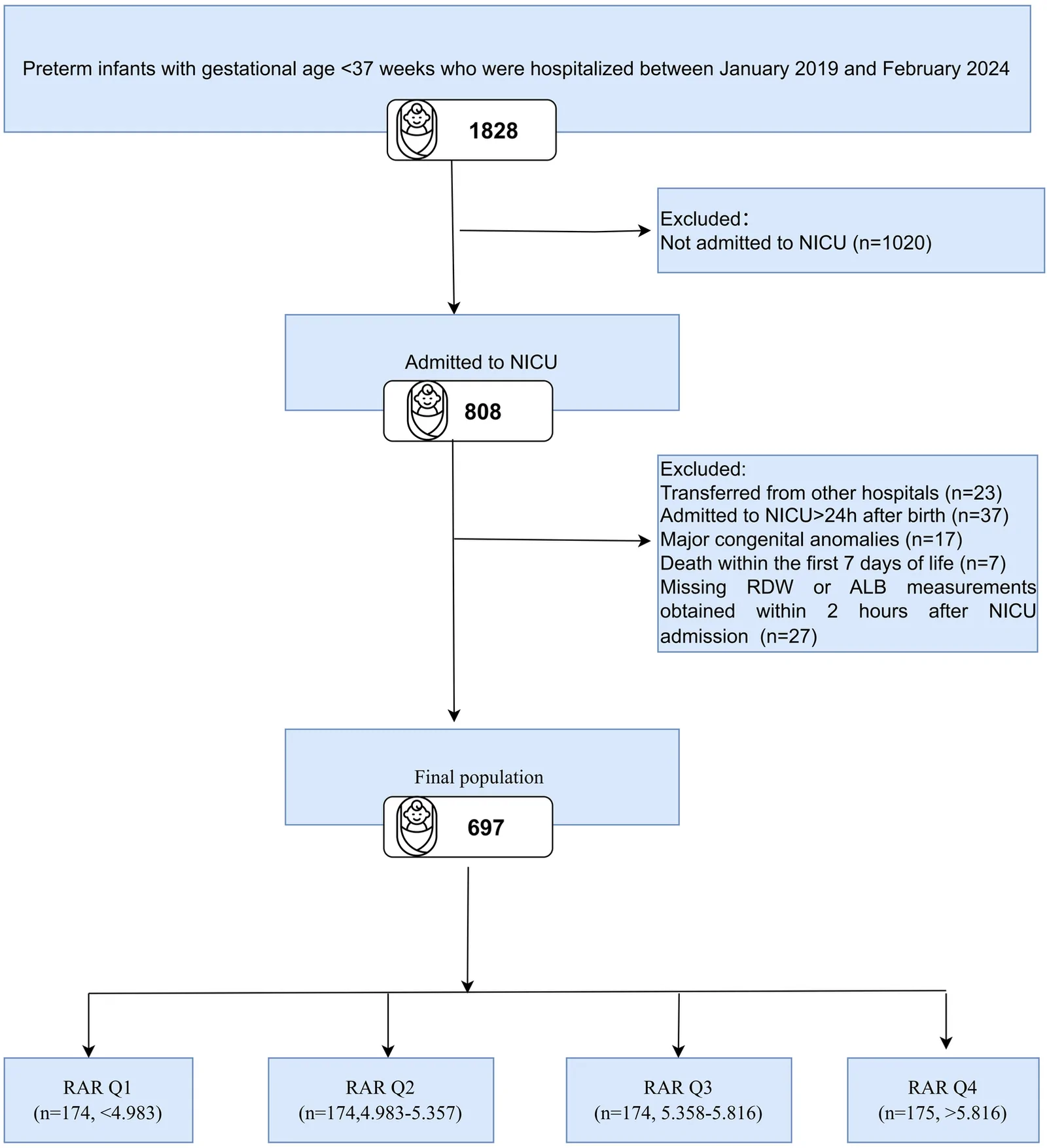
Flowchart of participant selection.

Preterm infants were included if they were born at Jiangmen Maternity and Child Health Care Hospital with a gestational age between 24⁰⁄₇ and 36⁶⁄₇ weeks and were directly admitted to the NICU from the delivery room within 24 h after birth. Exclusion criteria were as follows: (1) non-admission to the NICU; (2) transfer from another hospital; (3) admission to the NICU more than 24 h after birth; (4) major congenital anomalies (including major congenital heart disease, congenital airway or pulmonary malformations, gastrointestinal malformations, and other severe structural abnormalities); (5) death within the first 7 days of life; and (6) missing RDW or albumin measurements obtained within 2 h after NICU admission. Infants who received antibiotics for noninfectious indications were not excluded; all antibiotic exposures during hospitalization were recorded and analyzed. Because all exposures and outcomes were assessed during the index hospitalization, no infants were excluded because of incomplete follow-up (Figure 1).

### Measurement of RAR

Laboratory measurements were performed on admission. Blood for both complete blood count (including RDW) and serum chemistry (including albumin) was obtained from the same blood draw and collected within 2 h of NICU admission. Samples were typically drawn after initial stabilization on admission and before major therapeutic interventions whenever clinically feasible. ALB was measured by the bromocresol green method (Mindray BS-2000M, Mindray, Shenzhen, China), and RDW was determined via complete blood count using a Sysmex XN-series analyzer (Sysmex, Kobe, Japan) with impedance and scatter/fluorescence analysis. RAR was calculated as RDW (%) divided by ALB (g/dL) using these initial values obtained from the same blood sample.

### Outcome definition

The primary composite outcome comprised neonatal pneumonia and/or blood culture-proven sepsis during the NICU stay, excluding unconfirmed clinical sepsis. These infections typically occurred and were diagnosed several days after NICU admission, following the baseline RAR measurement.

Neonatal pneumonia was defined based on compatible clinical manifestations, radiographic findings, and laboratory evidence of infection, and antibiotic treatment duration ≥5 days was recorded only as treatment information and was not used as part of the diagnostic criterion. Tracheal aspirate cultures, when available, were recorded but were not required for diagnosis. In infants with underlying respiratory disorders (respiratory distress syndrome, evolving bronchopulmonary dysplasia, atelectasis, or pulmonary edema), pneumonia required superimposed clinical deterioration, new radiographic abnormalities, and laboratory evidence of infection beyond that expected from the underlying condition (7, 8).

Culture-proven sepsis was defined as isolation of a recognized pathogen from a peripheral blood culture obtained before antibiotic administration in the presence of compatible systemic manifestations. Infants could fulfill criteria for both pneumonia and sepsis; such overlapping cases were included in the composite outcome (9, 10). Due to data limitations, early-onset and late-onset infections could not be strictly differentiated; thus, all infection events occurring after the baseline RAR measurement during the NICU stay were recorded.

### Covariates

Potential confounders were selected *a priori* based on biological plausibility and evidence from the literature for association with both neonatal infection and RAR. These included infant factors [sex, GA in completed weeks, birth weight (g), small for gestational age (SGA) status, defined as birth weight <10th percentile for GA using local growth curves, and multiple gestation] (11), delivery characteristics [mode of delivery (vaginal vs. cesarean) and prelabor rupture of membranes] (12), maternal comorbidities [gestational hypertension diagnosed according to ACOG criteria (13) and gestational diabetes mellitus per IADPSG criteria (14)], and initial laboratory markers measured within 2 h of NICU admission, including metabolic parameters (pH, glucose, lactate) and organ function indices (ALT, creatinine).

### Statistical analysis

Continuous variables were assessed for normality. Data are presented as mean ± standard deviation (SD) for normally distributed variables, median (interquartile range, IQR) for non-normally distributed variables, and number (percentage) for categorical variables. The primary exposure, the RAR was analyzed as both a continuous variable and by study-derived quartiles (Q1: <4.983, Q2: 4.983–5.357, Q3: 5.358–5.816, Q4: ≥5.816). Group comparisons were performed using Student's t-test or one-way ANOVA for normally distributed continuous variables, the Kruskal–Wallis H test for non-normally distributed variables, and the chi-square (*χ*^2^) test for categorical variables. Missing data were minimal (5%) and handled by listwise deletion (Supplementary Table S1).

To assess the association between the RAR and the subsequent composite neonatal infection outcome, logistic regression analyses were conducted, with RAR evaluated both as a continuous variable and by quartiles. Univariate analyses were performed initially (Supplementary Table S2), and covariates for multivariable models were selected based on clinical relevance, prior literature, and univariate screening. Multicollinearity was checked using variance inflation factors (VIF >5, Supplementary Table S3). Two models were fitted: an unadjusted model and a multivariable-adjusted model controlling for sex, birth weight, GA, SGA status, gestational hypertension, prelabor rupture of membranes, multiple gestation, and initial laboratory values (pH, glucose, and lactate). Trend analyses across quartiles were performed to detect dose–response relationships. Hepatic (ALT) and renal (creatinine) markers were deliberately excluded from the primary model to avoid potential overadjustment, which could obscure the independent effect of RAR. Spearman rank correlation was also conducted to explore relationships among RDW, ALB, RAR, and neonatal infection status. Receiver operating characteristic (ROC) curve analyses were performed to assess the risk stratification performance of RDW, ALB, and RAR for the subsequent composite infection outcome. In a secondary exploratory analysis, ROC curves were also constructed to compare the relative discrimination of RAR, the CRP-to-albumin ratio (CAR), CRP, and PCT for infection risk stratification among infants with available biomarker measurements. Pairwise comparisons of AUCs were conducted using DeLong's test.

Nonlinear associations were assessed using restricted cubic splines (RCS) with three knots at the 10th, 50th, and 90th percentiles (15). When visual inspection of the spline curves suggested potential nonlinearity, a two-piecewise linear regression model was fitted to further explore possible inflection points.

Subgroup analyses were conducted stratified by GA (<32 weeks, 32–33weeks, and ≥34 weeks), birth weight (1,250 g vs. ≥ 1,250 g), SGA status, and multiple gestation. Participants were classified into low RAR (Q1–Q2) and high RAR (Q3–Q4) groups according to the median RAR value. Interactions were tested using likelihood ratio tests. To account for multiple testing, *P* values were adjusted using the Bonferroni correction, and adjusted *P* < 0.05 was considered statistically significant.

Sensitivity analyses were performed to assess the robustness of the main findings. Logistic regression models were repeated after additionally adjusting for hepatic (ALT) and renal (creatinine) function markers.

E-value analysis was conducted to quantify the minimum strength of an unmeasured confounder that would be required to fully explain the observed associations (16).

All statistical analyses were performed using R software (version 4.5.2; R Foundation for Statistical Computing, Vienna, Austria) and Free Statistics software (version 2.3; Beijing Free Clinical Medical Technology Co., Ltd., Beijing, China).

## Results

### Characteristics of the study population

This study included 697 preterm infants in the NICU from January 2019 to February 2024. Baseline characteristics by RAR quartiles are shown in Table 1. The cohort had a mean GA of 32.7 ± 2.4 weeks, a median GA of 33.0 weeks (IQR, 31.1–34.4 weeks), ranging from 25.29 to 36.86 weeks, and a mean birth weight of 1,810.3 ± 526.8 g. Compared with the lowest quartile (Q1), preterm infants in the highest quartile (Q4) had lower GA (32.1 ± 2.7 vs. 33.2 ± 2.1 weeks), and birth weight (1,657.9 ± 525.9 vs. 1,931.0 ± 549.6 g), higher prevalence of SGA status (21.1% vs. 14.4%), and a higher proportion of males (62.3% vs. 46.6%). RDW increased across quartiles (Q1: 15.7 ± 0.9%, Q4: 18.2 ± 2.4%), while ALB decreased (Q1: 3.4 ± 0.2 g/dL, Q4: 2.8 ± 0.3 g/dL). The prevalence of composite neonatal infection was significantly higher in Q4 than in Q1 (71.4% vs. 50.0%). Among infection events, neonatal pneumonia accounted for the majority of cases (423/448, including 9 cases with concurrent blood culture–proven sepsis), whereas isolated blood culture–proven sepsis represented a small proportion (25/448).

**Table 1:** Maternal and infant characteristics.

Variables	RAR	Q1 (<4.983)	Q2 (4.983–5.357)	Q3 (5.358–5.816)	Q4 (>5.816)	*P*-value
	Total (*n* = 697)	(*n* = 174)	(*n* = 174)	(*n* = 174)	(*n* = 175)	
**Maternal Information**						
Maternal age (years)	30.6 ± 5.0	30.2 ± 5.1	30.4 ± 5.0	30.8 ± 5.4	30.8 ± 4.8	0.766
Cesarean delivery, *n* (%)	463 (66.5)	105 (60.3)	116 (66.7)	120 (69)	122 (70.1)	0.300
Multiple birth, *n* (%)	161 (23.1)	27 (15.5)	38 (21.8)	48 (27.6)	48 (27.4)	0.022
Gestational hypertension, *n* (%)	50 (7.2)	10 (5.7)	12 (6.9)	12 (6.9)	16 (9.1)	0.661
Gestational diabetes mellitus, *n* (%)	214 (30.7)	62 (35.6)	44 (25.3)	51 (29.3)	57 (32.6)	0.185
Prelabor rupture of membranes, *n* (%)	204 (29.3)	60 (34.5)	49 (28.2)	52 (29.9)	43 (24.6)	0.232
**Infants' Information**						
Sex, *n* (%)						0.022
Female	308 (44.2)	93 (53.4)	77 (44.3)	72 (41.4)	66 (37.7)	
Male	389 (55.8)	81 (46.6)	97 (55.7)	102 (58.6)	109 (62.3)	
GA continuous (weeks)	32.7 ± 2.4	33.2 ± 2.1	32.9 ± 2.3	32.4 ± 2.3	32.1 ± 2.7	<0.001
GA continuous (weeks)	33.0 (31.1, 34.4)	33.3 (31.9, 34.7)	33.2 (31.4, 34.7)	32.9 (30.7, 34.0)	32.4 (30.4, 34.0)	<0.001
GA categorical (weeks), *n* (%)						0.001
<28	20 (2.9)	1 (0.6)	1 (0.6)	7 (4)	11 (6.3)	
28–31	224 (32.1)	43 (24.7)	53 (30.5)	63 (36.2)	65 (37.1)	
32–33	213 (30.6)	59 (33.9)	54 (31)	56 (32.2)	44 (25.1)	
≥34	240 (34.4)	71 (40.8)	66 (37.9)	48 (27.6)	55 (31.4)	
SGA status, *n* (%)	100 (14.3)	25 (14.4)	14 (8)	24 (13.8)	37 (21.1)	0.007
Birth weight (g)	1,810.3 ± 526.8	1,931.0 ± 549.6	1,882.1 ± 514.4	1,771.2 ± 476.3	1,657.9 ± 525.9	<0.001
RDW (%)	16.7 ± 1.8	15.7 ± 0.9	16.3 ± 1.2	16.8 ± 1.2	18.2 ± 2.4	<0.001
pH	7.3 ± 0.1	7.3 ± 0.1	7.3 ± 0.1	7.3 ± 0.1	7.3 ± 0.1	0.271
Glucose (mmol/L)	5.3 ± 1.8	5.2 ± 1.7	5.4 ± 1.7	5.6 ± 1.9	5.1 ± 1.8	0.165
Lactate (mmol/L)	2.1 (1.7, 2.8)	2.1 (1.7, 2.7)	2.1 (1.6, 2.6)	2.1 (1.8, 2.9)	2.1 (1.7, 3.0)	0.421
ALB (g/dL)	3.1 ± 0.3	3.4 ± 0.2	3.2 ± 0.2	3.0 ± 0.2	2.8 ± 0.3	<0.001
ALT (U/L)	5.6 (4.1, 7.4)	6.0 (4.4, 8.9)	5.8 (4.5, 7.3)	5.6 (3.9, 7.4)	5.3 (3.7, 6.6)	0.003
Creatinine (umol/L)	56.4 ± 16.7	55.7 ± 14.4	55.3 ± 14.7	56.5 ± 17.1	58.2 ± 19.9	0.377
Antibiotic therapy	11.5 ± 11.6	8.7 ± 10.0	11.2 ± 12.9	12.6 ± 11.2	13.5 ± 11.8	<0.001
Neonatal pneumonia, *n* (%)	423 (60.7)	83 (47.7)	106 (60.9)	116 (66.7)	118 (67.4)	<0.001
Isolated culture-proven sepsis, *n* (%)	25 (3.6)	4 (2.3)	5 (2.9)	9 (5.2)	7 (4)	0.486
Composite neonatal infection, *n* (%)	448 (64.3)	87 (50)	111 (63.8)	125 (71.8)	125 (71.4)	<0.001

RAR, red cell distribution width to albumin ratio (RDW (%)/albumin (g/dL)); GA, gestational age; SGA, small for gestational age; RDW, red cell distribution width; ALB, albumin; ALT, alanine Aminotransferase.

### Correlation patterns and exploratory ROC analysis of biomarkers

**Figure 2:**
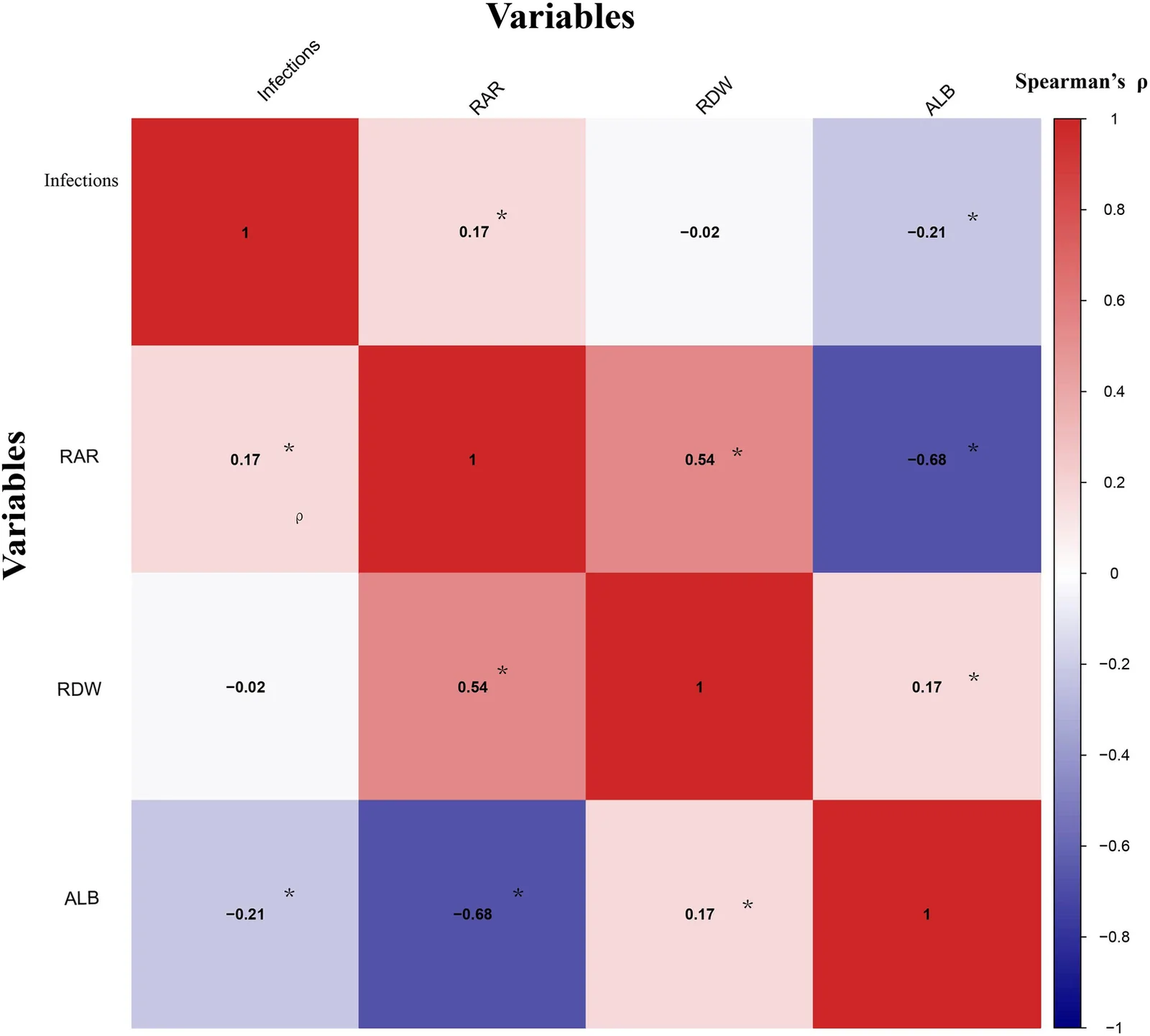
Spearman rank correlation matrix of RAR, RDW, ALB, and composite neonatal infection. Colors indicate correlation strength and direction (red: positive, blue: negative; intensity proportional to *ρ*). Numerical r values are shown in the matrix; *P* values are not displayed. Diagonal elements represent perfect self-correlation (*ρ*= 1). An asterisk (*) indicates statistical significance at *P* < 0.001.

**Figure 3:**
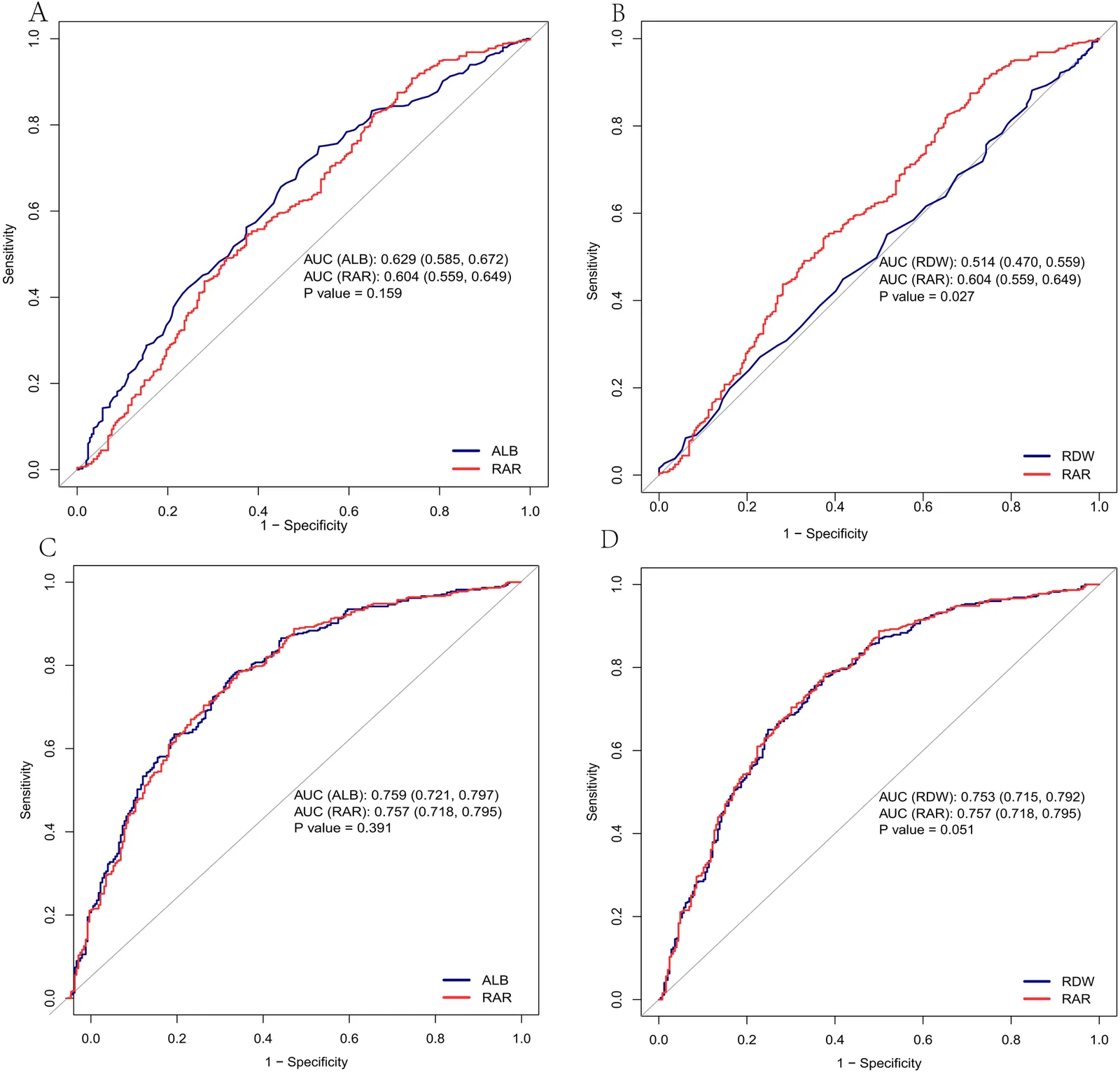
ROC curve analysis comparing individual indicators and multivariable-adjusted models for composite neonatal infection. **(A)** Unadjusted ROC curves comparing standalone ALB and RAR. **(B)** Unadjusted ROC curves comparing standalone RDW and RAR. **(C)** ROC curves comparing the multivariable-adjusted model incorporating ALB versus the model incorporating RAR. **(D)** ROC curves comparing the multivariable-adjusted model incorporating RDW versus the model incorporating RAR. Multivariable models were adjusted for sex, birth weight, GA, SGA status, gestational hypertension, prelabor rupture of membranes, multiple birth, pH, glucose, and lactate. *P*-values were calculated using DeLong's test to evaluate the differences between the respective areas under the curves (AUCs). Data in parentheses represent the 95% confidence intervals.

Spearman correlation analysis showed a moderate negative association between ALB and composite neonatal infection (*ρ*=–0.213, *P* < 0.001), while the RAR was weakly positively correlated with infection (*ρ*= 0.172, *P* < 0.001). RDW alone was not significantly associated with infection status (*ρ*=–0.024, *P* = 0.534) (Supplementary Table S4). Hierarchical clustering (Figure 2) highlighted distinct patterns among these biomarkers, with ALB and infection status forming a coherent cluster reflecting their inverse relationship. In exploratory single-biomarker ROC analyses, ALB showed the highest discrimination (AUC = 0.629), followed by RAR (AUC = 0.604), whereas RDW demonstrated limited performance (AUC = 0.514). No significant difference was observed between ALB and RAR (DeLong's *P* = 0.159), whereas RAR demonstrated a modestly higher discrimination than RDW (*P* = 0.027). In multivariable-adjusted models, ALB and RAR showed comparable performance and showed highly comparable performance to the model incorporating RDW (AUC = 0.759 vs. 0.757 vs. 0.753) (Figures 3A–D).

**Figure 4:**
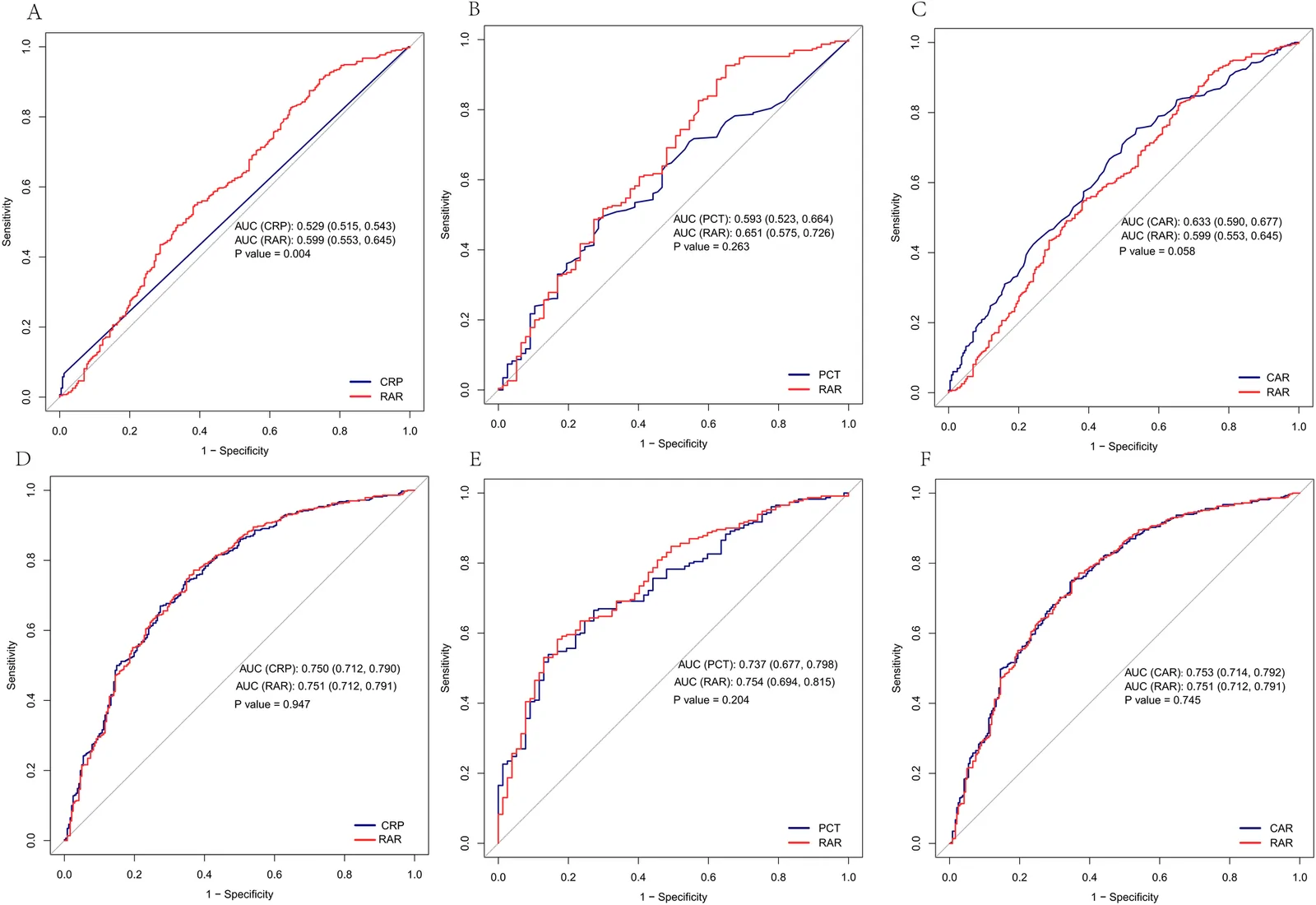
Comparison of the performance of RAR, CAR, CRP, and PCT in association with composite neonatal infection. **(A)** ROC curves comparing single-biomarker performance of RAR and CRP within the CRP subgroup (*n* = 676). **(B)** ROC curves comparing single-biomarker performance of RAR and PCT within the procalcitonin subgroup (*n* = 307). **(C)** ROC curves comparing the discriminatory performance of RAR and the CRP-to-albumin ratio (CAR) in the CRP subgroup (*n* = 676). **(D)** ROC curves comparing multivariable-adjusted models incorporating RAR versus CRP within the CRP subgroup. **(E)** ROC curves comparing multivariable-adjusted models incorporating RAR versus PCT within the procalcitonin subgroup. **(F)** ROC curves comparing multivariable-adjusted models incorporating RAR and CAR in the CRP subgroup. Multivariable models were adjusted for sex, birth weight, GA, SGA status, gestational hypertension, prelabor rupture of membranes, multiple birth, pH, glucose, lactate. Area under the curve (AUC) values are shown with 95% confidence intervals in parentheses. *P*-values were calculated using DeLong's test for comparing correlated ROC curves. The dotted line represents the diagonal reference line (AUC = 0.5).

Among infants with available CRP measurements, CAR yielded a numerically higher unadjusted AUC than RAR [0.633 (95% CI, 0.590–0.677) vs. 0.599 (95% CI, 0.553–0.645)], although the difference was not statistically significant (DeLong's *P* = 0.058). After multivariable adjustment, the two models showed nearly identical discrimination [AUC = 0.753 (95% CI, 0.714–0.792) vs. 0.751 (95% CI, 0.715–0.791); *P* = 0.745]. In the same subgroup, RAR showed a statistically higher unadjusted AUC than CRP(AUC = 0.599 vs. 0.529; *P* = 0.004), whereas, adjusted models achieved comparable discrimination (AUC = 0.751 vs. 0.750; *P* = 0.947). Similarly, RAR and PCT demonstrated comparable discrimination in both unadjusted and adjusted analyses (Figures 4A–F).

### Associations between RAR and composite neonatal infection

To assess the independent association, multivariable logistic regression analyses were performed. When analyzed as a continuous variable, RAR was not significantly associated with the composite infection outcome after adjustment [Odds ratios (OR) = 1.15, 95% confidence interval (CI): 0.92–1.44, *P* = 0.212]. In quartile-based analyses for the composite infection outcome, all higher RAR quartiles were significantly associated with increased odds compared with Q1, including Q2 (OR = 1.73, 95% CI: 1.08–2.79; *P* = 0.023), Q3 (OR = 2.33, 95% CI: 1.42–3.81; *P* = 0.001), and Q4 (OR = 2.02, 95% CI: 1.22–3.35; *P* = 0.006), with a significant trend across quartiles(*P* for trend = 0.002).

In analyses stratified by infection type, a similar pattern was observed for neonatal pneumonia. Compared with Q1, higher odds of pneumonia were observed in Q2 (OR = 1.65, 95% CI: 1.05–2.62), Q3 (OR = 2.01, 95% CI: 1.25–3.22), and Q4 (OR = 1.91, 95% CI: 1.17–3.10), with a significant overall trend (*P* for trend = 0.005). The magnitude of association varied across quartiles, with Q4 not exceeding Q3, indicating heterogeneity in effect estimates across exposure categories. For blood culture–proven sepsis, the number of events was limited (*n* = 25), effect estimates were directionally consistent with the composite outcome and pneumonia but did not reach statistical significance (OR = 1.33, 95% CI: 0.89–1.99, *P* = 0.158) (Table 2). Infection subtypes were not mutually exclusive, and neonatal pneumonia accounted for most events in the composite infection outcome.

**Table 2:** Associations between RAR and composite neonatal infection in the multiple logistic regression model. .

Variable	Total	Events, n(%)	Crude Model OR (95%CI)	*P*-value	Adjusted Model OR (95%CI)	*P*-value
Composite neonatal infection						
RAR (continuous)	697	448 (64.3)	1.34 (1.08–1.65)	0.007	1.15 (0.92–1.44)	0.212
RAR (Quartile)						
Q1	174	87 (50)	1(Ref)		1(Ref)	
Q2	174	111 (63.8)	1.76 (1.15–2.71)	0.010	1.73 (1.08–2.79)	0.023
Q3	174	125 (71.8)	2.55 (1.64–3.98)	<0.001	2.33 (1.42–3.81)	0.001
Q4	175	125 (71.4)	2.50 (1.61–3.89)	<0.001	2.02 (1.22–3.35)	0.006
*P* for trend				<0.001		0.002
Pneumonia						
RAR (continuous)	697	423 (60.7)	1.2 (0.99–1.45)	0.062	1.06 (0.87–1.31)	0.551
RAR (Quartile)						
Q1	174	83 (47.7)	1(Ref)		1(Ref)	
Q2	174	106 (60.9)	1.71 (1.12–2.62)	0.014	1.65 (1.05–2.62)	0.032
Q3	174	116 (66.7)	2.19 (1.42–3.38)	<0.001	2.01 (1.25–3.22)	0.004
Q4	175	118 (67.4)	2.27 (1.47–3.5)	<0.001	1.91 (1.17–3.10)	0.009
*P* for trend				<0.001		0.005
Blood culture–proven sepsis						
RAR (continuous)	697	25 (3.6)	1.39 (1.02–1.89)	0.039	1.33 (0.89–1.99)	0.158
RAR (Quartile)						
Q1	174	4 (2.3)	1(Ref)		1(Ref)	
Q2	174	5 (2.9)	1.26 (0.33–4.76)	0.736	1.10 (0.28–4.36)	0.891
Q3	174	9 (5.2)	2.32 (0.70–7.67)	0.169	1.74 (0.5–6.06)	0.387
Q4	175	7 (4)	1.77 (0.51–6.16)	0.369	1.03 (0.27–3.87)	0.969
*P* for trend				0.243		0.860

Crude Model: Unadjusted.

Adjusted Model: Adjusted for sex, birth weight, GA, SGA status, gestational hypertension, prelabor rupture of membranes, multiple birth, pH, glucose, lactate.

RAR, red cell distribution width to albumin ratio (RDW (%)/albumin (g/dL)); GA, gestational age; SGA, small for gestational age.

**Figure 5:**
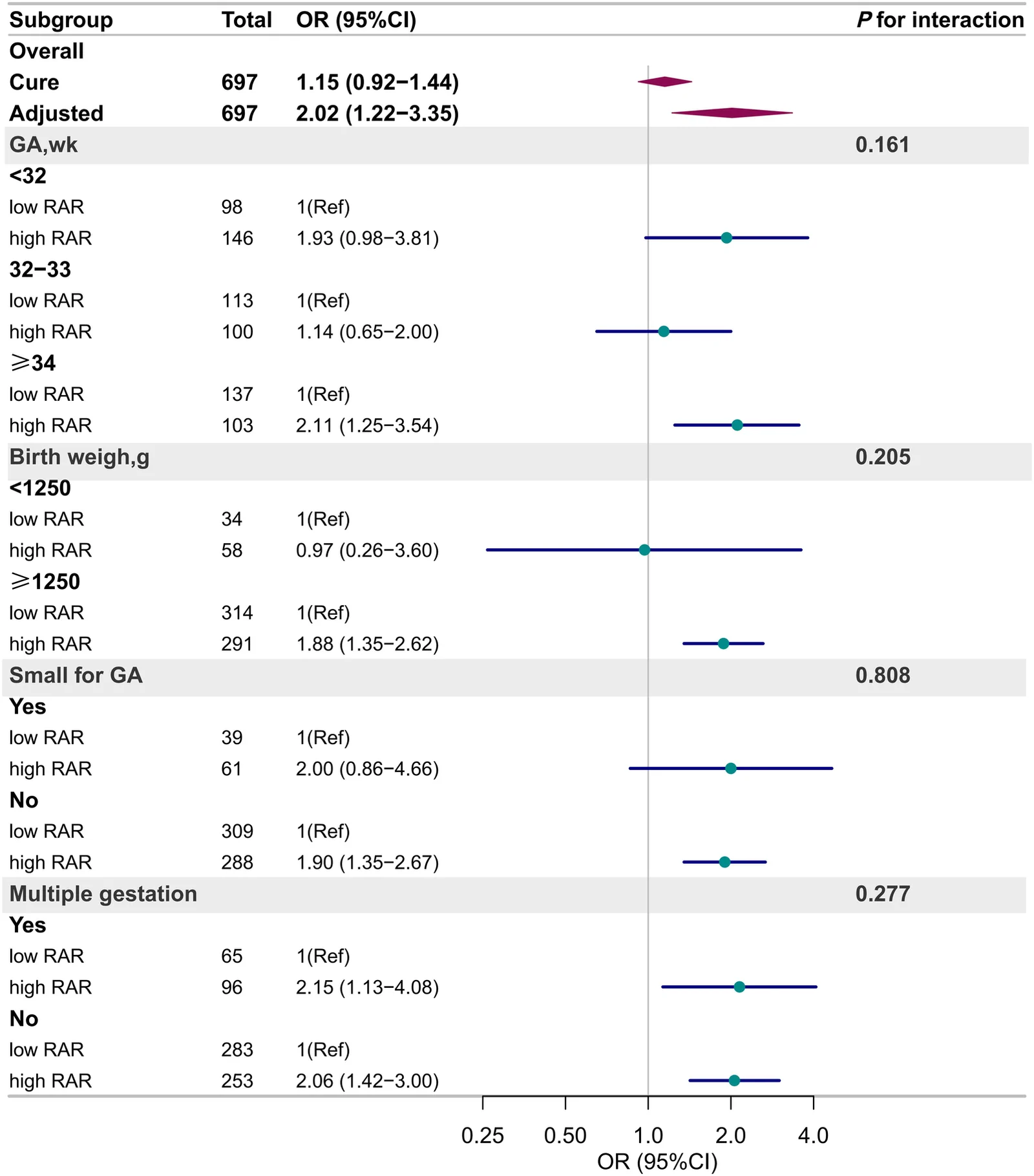
Forest plots of composite neonatal infection in different subgroups. Odds ratios (ORs) and 95% confidence intervals (CIs) were estimated from models adjusted for sex, birth weight, GA, SGA, gestational hypertension, prelabor rupture of membranes, multiple birth, pH, glucose, and lactate. Subgroup analyses were stratified by GA(<32 weeks, 32-33 weeks, ≥34 weeks), birth weight (<1,250 g and ≥1,250 g), SGA status, and multiple gestation. Participants were classified into low-RAR (Q1–Q2) and high-RAR (Q3–Q4) groups according to the median RAR value. No significant interactions were observed between RAR and any subgroup (all *P* for interaction >0.05). RAR: Red cell distribution width to albumin ratio (RDW (%)/albumin (g/dL)); GA: Gestational age; SGA: Small for gestational age.

Stratified analyses across key demographic and clinical subgroups showed generally consistent associations between higher RAR quartiles and infection risk, with no significant interactions after Bonferroni correction (all *P* for interaction > 0.005) (Figure 5; Supplementary Table S5).

### Dose-response relationship and non-linear association

**Figure 6:**
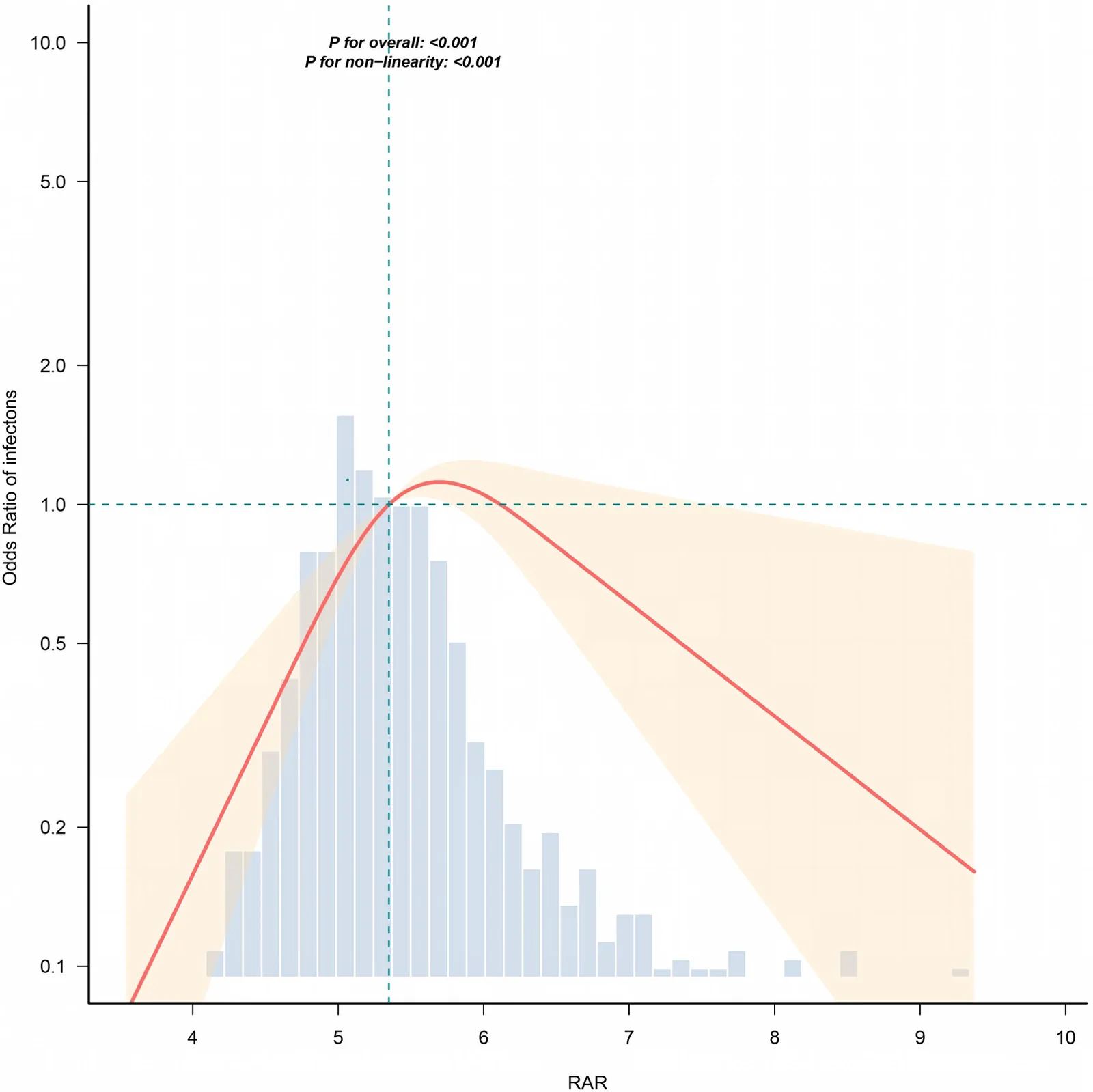
Restricted cubic spline curves of RAR and composite neonatal infection. The curve shows the adjusted dose–response relationship using restricted cubic splines with 3 knots, truncated at the 99.55th percentile. Models were adjusted for sex, birth weight, GA, SGA, gestational hypertension, prelabor rupture of membranes, multiple birth, pH, glucose, and lactate. Shaded areas represent the 95% confidence interval. *P* values indicate the overall association (*P* < 0.001) and non-linearity (*P* < 0.001). RAR: Red cell distribution width to albumin ratio (RDW (%)/albumin (g/dL)); GA: Gestational age; SGA: Small for gestational age.

Overall, higher RAR quartiles were associated with increased infection risk, with a non-linear relationship identified in subsequent analyses. Multivariable-adjusted RCS analyses indicated a nonlinear association between RAR levels and the risk of composite neonatal infection (*P* for nonlinearity < 0.001). An inflection point was identified at an RAR value of 5.65 (Figure 6). Below this threshold (RAR < 5.65), higher RAR was associated with a significantly increased risk of composite neonatal infection (*n* = 461, OR = 2.91, 95% CI: 1.59–5.31, *P* < 0.001). In contrast, above this threshold (RAR≥5.65), the association was not statistically significant (*n* = 227, OR = 0.62, 95% CI: 0.35–1.07, *P* = 0.090) (Table 3).

**Table 3:** Threshold effect analysis of the association between RAR and composite neonatal infection in NICU.

RAR	total	OR (95% CI)	*P*-value
RAR < 5.65	461	2.91 (1.59–5.31)	<0.001
RAR≥5.65	227	0.62 (0.35–1.07)	0.090
Likelihood Ratio test		-	<0.001

Odds ratios (ORs) and 95% confidence interval (CI) were derived from multivariable logistic regression models, adjusted for sex, birth weight, GA, SGA status, gestational hypertension, prelabor rupture of membranes, multiple birth, pH, glucose, and lactate.

RAR, red cell distribution width to albumin ratio [RDW (%)/albumin (g/dL)]; GA, gestational age; SGA, small for gestational age.

### Sensitivity and E-value analyses

Sensitivity analyses yielded consistent results (Supplementary Table S6). The positive association between higher RAR quartiles (Q3/Q4) and increased infection risk remained statistically significant in logistic regression models with additional adjustment for hepatic (ALT) and renal (creatinine) function markers. A significant positive trend across RAR quartiles was also observed in these models (all *P* for trend < 0.05). E-value analysis indicated that an unmeasured confounder would need to have a substantial effect (E-values ranging from 1.57 to 3.46 for point estimates) to fully explain the observed associations (Supplementary Table S7).

## Discussion

In this retrospective cohort study of preterm infants, we examined the association between admission RAR and composite neonatal infection. Higher RAR levels were associated with a greater likelihood of infection, particularly when analyzed in quartiles rather than as a continuous variable, suggesting a non-linear pattern. After multivariable adjustment, infants in the upper RAR quartiles had significantly higher odds of infection than those in the lowest quartile. RCS analyses further indicated a non-linear relationship with an apparent inflection point, below which higher RAR was associated with increased infection risk. These findings suggest that combining hematologic and nutritional indicators may provide additional information beyond either component alone.

The positive association between RAR and infection risk in preterm infants mirrors adult findings in sepsis (4, 17) and community-acquired bacteremia (18). This consistency supports RAR as a marker of physiological stress during infection. In our analysis, RAR showed a consistent association with infection outcomes, similar to observations in cardiovascular disease (19), suggesting that integrating inflammatory and nutritional status into a single index may better reflect underlying vulnerability. However, ROC analyses indicated that albumin alone demonstrated comparable discrimination and contributed substantially to the observed performance of RAR. The differences in discrimination among individual biomarkers were modest and should not be interpreted as clinically meaningful superiority of one marker over another. Therefore, RAR should be regarded as a complementary composite biomarker reflecting integrated biological processes rather than a standalone marker expected to outperform its individual components. Similarly, exploratory analyses demonstrated comparable discrimination between RAR and CAR, suggesting that these composite biomarkers may capture overlapping aspects of inflammation and nutritional status. Notably, the association between RAR and neonatal infection appeared to be primarily driven by neonatal pneumonia. Given the limited number of blood culture–confirmed cases, the sepsis subgroup was underpowered and should be interpreted as exploratory. Therefore, no definitive inference can be made regarding differences in associations across infection subtypes (20, 21).

A key observation is that the association between elevated RAR and neonatal pneumonia varies across exposure categories. Although significant associations were observed in multiple quartiles, the magnitude of association was not strictly increasing, with the highest quartile showing a relatively attenuated effect compared with Q3. Therefore, interpretation should focus on quartile-specific associations rather than assuming a strictly monotonic dose–response relationship.

The analysis further showed that the association between RAR and infection appeared non-linear, with a positive relationship observed when RAR was below 5.65, but no statistically significant increase beyond this threshold. This pattern should be interpreted cautiously rather than suggesting a potential biological “saturation effect,” this lack of significance more likely reflects that higher RAR levels may correspond to a potentially heterogeneous subgroup of preterm infants, in whom multiple concurrent pathophysiological processes could attenuate a clear association. It is also possible that this finding partly reflects reduced precision due to fewer observations at the distribution tail, potential overfitting of the spline model, or residual confounding. Therefore, RAR should be interpreted as a continuous marker rather than a threshold-based indicator. Importantly, this exploratory statistical threshold is not clinically actionable and requires robust prospective external validation before any clinical application can be considered.

Moreover, neonatal ALB is influenced by maternofetal transfer and postnatal fluid shifts (22, 23), suggesting that RAR may reflect developmentally relevant physiological stress beyond nutritional status alone. These features suggest that RAR may serve as an exploratory marker associated with subsequent pneumonia risk, providing complementary biological information alongside conventional inflammatory markers (24, 25).

From a clinical perspective, RAR may represent a complementary biomarker reflecting early physiological stress in preterm infants. In large ICU cohorts, elevated RAR improved prognostic accuracy when combined with established scoring systems such as SOFA, supporting its role as a complementary rather than standalone marker (26). In the present study, RAR demonstrated discrimination comparable to albumin alone. Although albumin showed numerically higher discrimination than RAR, the difference was not statistically significant, suggesting that hypoalbuminemia may be a major contributor to the observed association between RAR and neonatal infection. Nevertheless, as a composite marker integrating RDW and albumin, RAR may reflect both nutritional status and systemic stress. Accordingly, RAR should be considered a complementary biomarker rather than a replacement for established clinical assessment or conventional inflammatory markers. However, RAR should complement—not replace—gestational age, clinical signs, and traditional inflammatory markers. Future studies should assess its incremental risk stratification value alongside established biomarkers and determine whether targeted interventions for high-RAR infants improve outcomes (27).

Several limitations warrant consideration. First, the retrospective single-center design may limit generalizability and introduce residual confounding, although E-value analyses were performed. Second, some subgroup analyses were underpowered, particularly for culture-proven sepsis. For the isolated blood culture–proven sepsis subgroup (*n* = 25), limited statistical power precludes definitive inference and results should be interpreted as exploratory and inconclusive. Third, although neonatal infection was defined as a composite outcome, subgroup analyses indicated that the association was mainly observed in pneumonia cases. Moreover, our clinical and radiographic diagnostic criteria for pneumonia are inherently broader than strict microbiological culture-confirmed criteria, which likely contributed to the relatively high incidence of pneumonia observed in our cohort. Fourth, although secondary exploratory analyses indicated that admission RAR demonstrated discriminative performance comparable to CAR, CRP and PCT, these biomarkers—particularly procalcitonin—were not uniformly available across all infants due to the retrospective design. Therefore, the incremental clinical value of RAR beyond established inflammatory markers could not be comprehensively assessed in the full cohort. Fifth, there is a notable temporal gap between the assessment of RAR (within the first 2 h of life) and the clinical onset of pneumonia, which typically developed several days later. While baseline RAR may reflect intrauterine inflammatory exposure or baseline immune dysregulation, its predictive performance over time could be confounded by postnatal factors in the NICU, such as mechanical ventilation, respiratory support, and nosocomial exposures. Therefore, baseline RAR is best understood as an exploratory risk marker of systemic susceptibility rather than an immediate diagnostic tool for late-onset pulmonary infections. Future prospective multicenter studies are warranted to validate these findings and to further assess the value of RAR in comparison with, or in combination with, conventional inflammatory markers.

## Conclusion

In this retrospective cohort of preterm infants, a higher admission RAR was associated with the composite neonatal infection outcome, and this association was primarily driven by neonatal pneumonia, whereas no significant association was observed for culture-proven sepsis. As a composite marker reflecting both inflammatory and nutritional status, RAR may serve as a complementary marker for early clinical assessment. However, its performance was comparable to albumin without evidence of superiority over established inflammatory markers. Prospective multicenter studies are warranted to validate these findings.

## Data Availability

The raw data supporting the conclusions of this article will be made available by the authors, without undue reservation.
