# Supplementary material for: Admission red cell distribution width–to–albumin ratio and risk of neonatal pneumonia or culture-proven sepsis in preterm infants: a retrospective cohort study
Source: Front Pediatr. 2026 Aug 19;14:1855210. doi: 10.3389/fped.2026.1855210 (PMC13534139; doi:10.3389/fped.2026.1855210)
Supplement: Supplementary file 1 [file Table1.docx]

**Supplement Material**

**Table S1 Missing Data Frequency and Percentage in the Study Cohort.**

**Table S2 Univariate logistic regression analysis of composite neonatal infection in NICU.**

**Table S3 Assessment of covariates using change-in-estimate and variance inflation factor.**

**Table S4 Correlations among RAR, RDW, albumin, and colmpostie neonatal infection.**

**Table S5 *P* for interaction in subgroup analyses of the association between RAR and composite neonatal infection after Bonferroni correction.**

**Table S6 Sensitivity analyses of the association between RAR and compostie neonatal infection including ALT and creatinine as covariates in a multivariable logistic regression model.**

**Table S7 E-values for the Associations of RAR and composite neonatal infection.**

**Table S1 Missing data frequency and percentage in the study cohort.**

| **Variable** | **Missing Frequency** | **Missing percentage(%)** |
| --- | --- | --- |
| Creatinine (umol/L) | 1 | 0.14 |
| Glucose(mmol/L) | 2 | 0.29 |
| Lactate(mmol/L) | 1 | 0.14 |
| CRP (mg/L) | 21 | 3.01 |
| PCT (ng/mL) | 390 | 55.95 |

Note:

CRP:C-reactive protein; PCT:Procalcitonin.

**Table S2 Univariate logistic regression analysis of composite neonatal infection in NICU.**

| **Variable** | **OR (95%CI)** | ***P-*value** |
| --- | --- | --- |
| Sex | 1.11 (0.81-1.51) | 0.528 |
| GA continuous (weeks) | 0.71 (0.65-0.77) | <0.001 |
| SGA status | 1.09 (0.7-1.71) | 0.697 |
| Birth weight (g) | 1 (1-1) | <0.001 |
| Maternal age (years) | 1 (0.97-1.03) | 0.832 |
| Cesarean delivery | 0.71 (0.51-1) | 0.048 |
| Multiple birth | 0.53 (0.37-0.76) | 0.001 |
| Gestational hypertension | 1.63 (0.85-3.14) | 0.14 |
| Gestational diabetes mellitus | 1.07 (0.77-1.51) | 0.675 |
| Prelabor rupture of membranes | 1.23 (0.87-1.74) | 0.233 |
| RDW | 0.97 (0.89-1.06) | 0.523 |
| pH | 0.17 (0.03-1.05) | 0.057 |
| Glucose (mmol/L) | 1.14 (1.04-1.24) | 0.006 |
| Lactate (mmol/L) | 1.01 (0.91-1.12) | 0.877 |
| ALB (g/dL) | 0.88 (0.83-0.92) | <0.001 |
| RAR [%/(g/dl)] | 1.34 (1.08-1.65) | 0.007 |

Note:
RAR: Red cell distribution width to albumin ratio (RDW (%) / albumin (g/dL)); GA: Gestational age; SGA: Small for gestational age; RDW: Red cell distribution width; ALB: Albumin; ALT: Alanine Aminotransferase.

**Table S3 Assessment of covariates using change-in-estimate and variance inflation factor.**

| **Covariate** | **VIF** |
| --- | --- |
| Sex | 1.09 |
| GA continuous (weeks) | 3.58 |
| SGA status | 1.92 |
| Birth weight (g) | 3.84 |
| Ph | 1.17 |
| Lactate (mmol/L) | 1.08 |
| Glucose (mmol/L) | 1.11 |
| Maternal age (years) | 1.11 |
| Prelabor rupture of membranes | 1.05 |
| Gestational diabetes mellitus | 1.09 |
| Gestational hypertension | 1.08 |
| Multiple birth | 1.16 |

Note:
VIF: Variance inflation factor; GA: Gestational age; SGA: Small for gestational age; ALB: Albumin; ALT: Alanine Aminotransferase.

**Table S4 Correlations among RAR, RDW, albumin, and composite neonatal infection.**

| **Variable** | **RAR[(ρ (*P*-value)]** | **RDW[(ρ (*P*-value)]** | **ALB[(ρ (*P*-value)]** | **Neonatal infections[(ρ (*P*-value)]** |
| --- | --- | --- | --- | --- |
| RAR | 1 | 0.542 (<0.001) | −0.679 (<0.001) | 0.172 (<0.001) |
| RDW | 0.542 (<0.001) | 1 | 0.175 (<0.001) | −0.024 (0.534) |
| ALB | −0.679 (<0.001) | 0.175 (<0.001) | 1 | −0.213 (<0.001) |
| Composite neonatal infection | 0.172 (<0.001) | −0.024 (0.534) | −0.213 (<0.001) | 1 |

Note:
RAR: Red cell distribution width to albumin ratio (RDW (%) / albumin (g/dL)) ; RDW: Red cell distribution width;ALB: Albumin.

**Table S5 *P* for interaction in subgroup analyses of the association between RAR and composite neonatal infection after Bonferroni correction.**

| **Subgroup variable** | **Unadjusted *P* for interaciton** | **Adjusted *P* for interaciton** |
| --- | --- | --- |
| GA-stratified | 0.161 | 0.644 |
| Birth weight-stratified | 0.205 | 0.82 |
| SGA status-stratified | 0.808 | 1.00 |
| Multiple birth-stratified | 0.277 | 1.00 |

NOTE:

Bonferroni correction was applied to control the family-wise error rate across four subgroup comparisons. The adjusted significance threshold was set at α = 0.0125 (0.05/4). After Bonferroni adjustment, no subgroup effect reached the predefined significance threshold.

**Table S6 Sensitivity analyses of the association between RAR and composite neonatal infection including ALT and creatinine as covariates in a multivariable logistic regression model.**

| **Variable** | **Total** | **Infections, n(%)** | **Crude Model OR (95%CI)** | ***P*-value** | **Adjusted Model OR (95%CI)** | ***P*-value** |
| --- | --- | --- | --- | --- | --- | --- |
| RAR (continuous) | 697 | 448 (64.3) | 1.34 (1.08-1.65) | 0.007 | 1.18 (0.93-1.48) | 0.167 |
| RAR (quartile) |  |  |  |  |  |  |
| Q1 | 174 | 87 (50) | 1(Ref) |  | 1(Ref) |  |
| Q2 | 174 | 111 (63.8) | 1.76 (1.15-2.71) | 0.010 | 1.77 (1.10-2.85) | 0.020 |
| Q3 | 174 | 125 (71.8) | 2.55 (1.64-3.98) | <0.001 | 2.38 (1.45-3.91) | 0.001 |
| Q4 | 175 | 125 (71.4) | 2.50 (1.61-3.89) | <0.001 | 2.09 (1.25-3.47) | 0.005 |
| *P* for trend |  |  |  | <0.001 |  | 0.002 |

Note:

Crude Model: Unadjusted.

Adjusted Model: Adjusted for sex, birth weight, GA, SGA status, gestational hypertension, prelabor rupture of membranes, multiple birth, pH, glucose, lactate, ALT, creatinine.

RAR: Red cell distribution width to albumin ratio (RDW (%) / albumin (g/dL)); GA: Gestational age; SGA: Small for gestational age; ALT: Alanine Aminotransferase.

**Table S7 E-values for the Associations of RAR and composite neonatal infection.**

| **Variable** | **E-value for point estimate** | **E-value for lower CI bound** |
| --- | --- | --- |
| RAR (continuous) | 1.57 | 1.00 |
| RAR (Quartile) | 3.46 | 1.74 |
